# Determinants of tooth loss among adolescents (15–19 years) from Minas Gerais, Brazil: a multilevel analysis

**DOI:** 10.1590/1807-3107bor-2025.vol39.094

**Published:** 2025-09-08

**Authors:** Thiago Peixoto da MOTTA, Jessica Klockner KNORST, Ana Clara Valadares da SILVEIRA, Rafaela da Silveira PINTO, Débora Guedes da MOTA, Mauro Henrique Nogueira Guimarães de ABREU, Fabiana VARGAS-FERREIRA

**Affiliations:** (a) Universidade Federal de Minas Gerais – UFMG, School of Dentistry, Department of Community and Preventive Dentistry, Belo Horizonte, MG, Brazil.; (b) Universidade Federal de Santa Maria – UFSM, School of Dentistry, Department of Stomatology, Santa Maria, RS, Brazil.; (c) Universidade Federal de Minas Gerais – UFMG, School of Dentistry, Department of Community and Preventive Dentistry, Belo Horizonte, MG, Brazil.

**Keywords:** Adolescent, Social Determinants of Health, Multilevel Analysis, Tooth Loss

## Abstract

In this cross-sectional study, carried out in 2012, we assessed factors associated with tooth loss among adolescents from Minas Gerais, Brazil, utilizing data from a secondary database. Individual and local-level variables were selected to represent health determinants. Individual covariates included sex, age, skin color, maternal education, household income, use of dental services, and self-perceived need for dental care. The contextual variables included illiteracy, unemployment, income, primary health care coverage, dental specialty centers, and oral health team coverage. Multilevel logistic regression models were used to examine the relationship between contextual and individual variables and the outcome (STATA version 16.0) – odds ratio (OR) and 95% confidence interval (CI). The prevalence of tooth loss was 12.4%. Female individuals were 40% more likely to experience the outcome (OR: 1.40; 95%CI: 1.01–1.98). Increased age was associated with greater tooth loss (OR: 1.16; 95%CI: 1.03-1.31). Low maternal education (OR: 1.72; 95%CI: 1.13–2.61), low household income (OR: 1.71; 95% CI 1.09-2.67), and self-perceived dental needs (OR: 2.94; 95% CI 1.97-4.39) were also associated with the outcome. Regular dental visits reduced the likelihood of tooth loss by 38% (OR: 0.62; 95 CI 0.44–0.87). Larger tooth loss was observed in municipalities with higher illiteracy rates (OR: 1.04; 95%CI: 1.01–1.08). Municipalities with larger dental specialty centers were associated with the outcome (OR: 0.58; 95%CI: 0.37–0.92). Contextual and individual factors influenced tooth loss in adolescents from Minas Gerais, Brazil, but socioeconomic status was the main determinant.

## Introduction

Adolescence, defined as the developmental period between the ages of 10 and 19 years, is characterized by psychosocial changes and exposure to risk situations^1^ and behaviors. In addition, tooth loss is a progressive condition that reflects the oral health history of individuals and their access to health services.^2,3^ This outcome is considered a marker of both oral health and social inequality.^3^ The literature has shown that tooth loss has a negative impact on oral health-related quality of life.^4,5^

Most studies on adolescents evaluate tooth loss in terms of clinical aspects and/or socioeconomic issues at the individual level. Generally, adolescents with worse socioeconomic backgrounds at an individual level are more exposed to risk factors for oral health problems.^1,6-8^ The investigation of factors associated with oral health outcomes is particularly important during adolescence, as parental and/or caregiver supervision decreases during this phase, and oral health self-care may be neglected. Consequently, adolescents may become more vulnerable to oral health problems.

Social inequalities are characterized by differences in oral health outcomes among adolescents, which are influenced by household income. These disparities affect access to and the use of oral health care.^9^

Research on adolescent oral health has traditionally focused on the most prevalent oral diseases, such as dental caries and periodontal diseases. However, contemporary literature has explored other outcomes, including malocclusions, aesthetic concerns, dissatisfaction with dental appearance, purposes for using dental services, oral health-related quality of life, dental pain, and tooth loss.

Oral conditions, including tooth loss, are linked to socioeconomic status, oral health history, and access to healthcare services.^1,2^ Contextual variables are essential for understanding and analyzing the level of development and well-being of a society, as well as the coverage and utilization of healthcare services within a community, city/town, or country. The use of multilevel analysis is essential for assessing health outcomes, thus allowing for inferences at the contextual and individual levels.^10^

This study aimed to assess factors associated with tooth loss among adolescents (15–19 years) from Minas Gerais, Brazil. Our working hypothesis is that both individual and contextual variables influence tooth loss among adolescents in the state of Minas Gerais, Brazil.

## Methods

This is a cross-sectional study conducted using secondary data obtained from the Minas Gerais Oral Health Study (SB Minas Gerais), carried out in 2012, to evaluate the oral health status of residents in the state of Minas Gerais, located in outheastern Brazil. The methodology of the 2010 national survey was used as reference for the collected indices: age, random selection of the municipalities, census tracts, households, and individual data.^12^

SB Minas Gerais was a state-level survey, representative of the state of Minas Gerais, that included 57 out of 853 municipalities, which were grouped into three broad domains: Capital, Inner Region I, and Inner Region II, based on the “local allocation factor” used to distribute healthcare funds. Inner Region I included more autonomous and less vulnerable municipalities, while Inner Region II encompassed more vulnerable and less autonomous municipalities. The study followed the established ethical guidelines and was approved by the Research Ethics Committee of the Pontifical Catholic University of Minas Gerais, Brazil.

The sample size for the capital was determined based on the prevalence and severity of dental caries in 2003.^4^ The same methodological approach was used for municipalities located in the inner region of the state, but the prevalence and severity of dental caries were based on data from the SBBrasil 2010 survey for the southeastern region.^12^ A minimum sample size of 750 individuals was estimated for the 15–19 age group; however, 1,202 adolescents were included in the study.

The examiners were trained and calibrated, yielding an inter-examiner agreement (Cohen’s kappa) greater than 0.65. Examinations were conducted in well-lit environments, following World Health Organization (WHO) guidelines.^11^ Oral conditions (dental caries, periodontal disease, dental occlusion, dental trauma, fluorosis, and edentulism) were assessed, and questionnaires were applied to assess socioeconomic factors, utilization of dental services, and need for dental treatment. All the information are available.^12^

For this study, the outcome variable — tooth loss — was derived from the “missing” component of the DMFT index. Individual variables included sex, age, skin color, maternal education, household income, utilization of dental services, and self-perceived need for dental care. All data were collected through interviews.

Contextual variables referred to characteristics of the regions where participants resided, with potential influence on the outcome. These contextual variables included unemployment, illiteracy, income less than half the minimum wage, primary health care (PHC) coverage, oral health team coverage in PHC, and availability of dental specialties centers. Data on contextual variables were collected from official information systems. All these variables are displayed in Table 1.

**Table 1:** Description of independent variables according to the level of analysis involving adolescents (n = 1,202), State of Minas Gerais, Brazil, 2012.

Variables	Description	Source
Sex	Male–Female	SB Minas Gerais 2012
Household income	Up to R$1,500 (Brazilian currency) (≤ 2BMW) and More than R$1,500 (>2BMW)	SB Minas Gerais 2012
Skin color	Self-reported skin color; a dichotomous variable was created from five original categories (white or non-white).	SB Minas Gerais 2012
Age	15–16 / 17–18 years	SB Minas Gerais 2012
Maternal education	Dichotomous: < 8 or ≥ 8 years	
Self-perception of dental needs	No need (healthy crown and root)	SB Minas Gerais 2012
	One surface restoration	
	Two or more surface restorations	
	Prosthetic crown needed for any reason	
	Dental facet	
	Pulp treatment and restoration	
	Tooth extraction	
	White spot treatment	
	Sealant	
Use of dental attendance	Dichotomous: regular user or non-regular user	SB Minas Gerais 2012
Illiteracy	Percentage (%) of individuals who cannot read or write and have no language proficiency in the total resident population in the minimum age range in a geographic space within the considered year	Census 2010 by DATASUS
		http://tabnet.datasus.gov.br/cgi/deftohtm.exe?ibge/censo/cnv/alfmg
		For more information (in Portuguese), go to
		http://tabnet.datasus.gov.br/cgi/ibge/censo/Taxa_Analfabetismo.pdf
Unemployment	Percentage (%) of economically active individuals unemployed during the reference week in a geographic space within the considered year	Census 2010 by DATASUS
		http://tabnet.datasus.gov.br/cgi/deftohtm.exe?ibge/censo/cnv/desemprmg
		For more information (in Portuguese), go to
		http://tabnet.datasus.gov.br/cgi/ibge/censo/Taxa_Desemprego.pdf
Half the BMMW	Percentage (%) of residents with monthly household income per capita up to half the Brazilian monthly minimum wage in a geographic space within the considered year	Census 2010 by DATASUS
		http://tabnet.datasus.gov.br/cgi/deftohtm.exe?ibge/censo/cnv/pobrezamg
		For more information (in Portuguese), go to
		http://tabnet.datasus.gov.br/cgi/ibge/censo/Prop_Pessoas_Baixa_Renda.pdf
Oral health team coverage	Percentage (%) of population covered by oral health teams	DATASUS / Ministry of Health, data for 2012.
		https://egestorab.saude.gov.br/paginas/acessoPublico/relatorios/relHistoricoCoberturaSB.xhtml
		For more information (in Portuguese), go to
		https://egestorab.saude.gov.br/paginas/acessoPublico/relatorios/nota_tecnica/nota_metodologica_SB.pdf
Primary health care coverage	Percentage (%) of the population covered by primary health care teams	DATASUS / Ministry of Health, data for 2012.
		https://egestorab.saude.gov.br/paginas/acessoPublico/relatorios/relHistoricoCoberturaAB.xhtml
		For more information (in Portuguese), go to
		https://egestorab.saude.gov.br/paginas/acessoPublico/relatorios/nota_tecnica/nota_tecnica_relatorio_de_cobertura_AB.pdf
Dental specialties centers	Percentage (%) of oral health facilities of the SUS system that are part of the National Registry of Health Establishments (CNES) and are classified as specialized clinics or specialty ambulatories.	Presence of dental specialties center, (2012 data). Data provided by the oral health coordination of the State Department of Health of Minas Gerais
		For more information (in Portuguese), go to
		https://www.gov.br/saude/pt-br/composicao/saps/brasil-sorridente/legislacao/ceo/portarias-de-habilitacoes
		http://cnes2.datasus.gov.br/Mod_Ind_Habilitacoes.asp?VEstado=31&VMun=&VComp=00&VTipo=H (see 0403, 0404, and 0405).

* $ Brazilian Real=$ 0.50 USD (Jul 2012); BMMW: Brazilian monthly minimum wage.

Multilevel logistic regression models were used to evaluate the relationship between contextual and individual variables and tooth loss, considering fixed effects with random intercepts. Additionally, the analysis was conducted based on a contextual framework (Figure) adapted from the World Health Organization. All analyses were performed using STATA 16 software.

**Figure:**
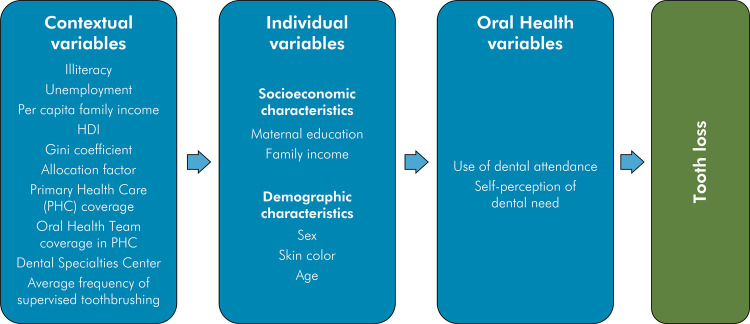
Theoretical framework for tooth loss in adolescents.^13^

Unadjusted and adjusted multilevel logistic regression analyses were used to evaluate the relationship between contextual and individual variables and tooth loss. The multilevel model used fixed effects with random intercepts, in which adolescents (1st level) were nested within 57 municipalities (2nd level). Our analysis was based on a theoretical framework developed by the Commission on Social Determinants of Health and considered three models: Model 1 (“empty model”); Model 2 (“contextual model”), including only contextual variables; and Model 3 (“full model”), which included both contextual and individual variables. The hierarchy of variables (in which contextual variables influence individual variables) was considered. Variables with a p ≤ 20 in the unadjusted analysis were considered for the adjusted models. In all models, the quality of fit was measured using deviance (-2 log-likelihood) and the odds ratio (OR). The results were presented as odds ratios (OR) and 95% confidence intervals (95%CI).

## Results

The study included 1,202 adolescents aged 15 to 19 years. Most participants were girls (55.3%), non-white (59.6%), and from households with an income ≤ 2BMW (57.7%). The prevalence of untreated dental caries was 39.8% (Table 2).

**Table 2:** Descriptive analysis of individual variables for the sample of adolescents (n = 1,202), State of Minas Gerais, Brazil, 2012.

Variables	n (%)
Sex	
Male	533 (44.7)
Female	669 (55.3)
Age (years)	
15	294 (24.5)
16	252 (21.0)
17	224 (18.6)
18	248 (20.6)
19	184 (15.3)
Skin color	
White	469 (40.4)
Non-white	733 (59.6)
Maternal education*	
> 8 years of formal education	941 (78.4)
≤ 8 years of formal education	260 (21.6)
Household income	
> 2 BMW	402 (42.3)
< 2 BMW	742 (57.7)
Use of dental services*	
Regular user	618 (51.6)
Non-regular user	578 (48.4)
Self-perception of dental needs	
No	619 (53.4)

*Missing values for some variables.

The prevalence of the outcome was 12.4%. Table 3 shows the unadjusted associations of contextual and individual variables with the outcome. The main finding was that increased age (OR 1.15; 95%CI 1.03-1.30) was associated with tooth loss. In addition, adolescents whose mothers had a low level of education (OR 2.13; 95%CI: 1.42-3.20) and who were from low-income families (OR 2.30; 95%CI 1.49-3.53) were more likely to experience tooth loss. Self-perceived dental needs (OR 3.62; 95%CI 2.44-5.37) were associated with tooth loss.

**Table 3:** Unadjusted (a) and adjusted (b) association between contextual and individual variables and outcome. State of Minas Gerais, Brazil, 2012.

Variables	OR^a^ (95% CI)	Model 1 (empty model)	Model 2 (contextual model)	Full model
		OR^b^ (95% CI)	OR^b^ (95% CI)	OR^b^ (95% CI)
Contextual variables				
Half the minimum wage	1.00 (0.99–1.00)		1.00 (0.99–1.00)	1.00 (0.99–1.00)
Illiteracy	1.05 (1.03–1.07)		1.04 (1.01–1.08)	1.04 (1.01–1.07)
Primary health care coverage	1.00 (0.99–1.00)		0.98 (0.97–1.01)	0.99 (0.98–1.00)
Oral health team coverage	1.00 (0.99–1.01)		1.00 (0.99–1.01)	0.99 (0.99–1.00)
Dental specialties center	0.44 (0.30–0.66)		0.58 (0.37–0.92)	0.54 (0.33–0.86)
Individual variables				
Sex				
Male	1.00			1.00
Female	1.33 (0.95–1.87)			1.40 (1.01–1.98)
Age (years)	1.15 (1.03–1.30)			1.16 (1.03–1.31)
Skin color				
White	1.00			
Non-white	1.01 (0.72–1.43))			
Maternal education				
8 years of formal education	1.00			1.00
< 8 years of forma education	2.13 (1.42–3.20)			1.72 (1.13–2.61)
Household income in R$				
> 2 BMW	1.00			1.00
≤ 2 BMW	2.30 (1.49–3.53)			1.71 (1.09–2.67)
Use of dental services				
Regular user	1.00			1.00
Self-perception of dental needs				
No	1.00			1.00
Yes	3.62 (2.44–5.37)			2.94 (1.97–4.39)

OR: odds ratio; CI: confidence interval; SMB: Brazilian minimum wage; Model 1: empty model, b Model 2: contextual variables; Model 3: adjusted for contextual and individual variables – variables not included and/or not associated with the outcom.e. Random component (deviance = (-2 loglikelihood). Empty model (900.7295); Contextual model (876.4025); Full model (781.2230).

The results of the adjusted multilevel logistic regression are shown in Table 3. Female individuals were 40% more likely to experience tooth loss (OR 1.40; 95%CI 1.01-1.98). Increased age (OR 1.16; 95%CI 1.03-1.31), low maternal education (OR 1.72; 95%CI 1.13–2.61), low household income (OR 1.71; 95%CI: 1.09–2.67) and self-perceived dental needs (OR 2.94; 95%CI: 1.97–4.39) were associated with tooth loss. Regular dental visits reduced the chance of having tooth loss by 38% (OR 0.62; 95%CI: 0.44–0.87). A higher prevalence of tooth loss was found in municipalities with larger illiteracy rates (OR 1.04; 95%CI: 1.01–1.08), whereas those municipalities with greater availability of dental specialty centers showed lower rates of tooth loss (OR 0.58; 95%CI: 0.37–0.92).

## Discussion

Our findings show that unfavorable contextual and individual variables are related to tooth loss in adolescents from the state of Minas Gerais. Municipalities with higher illiteracy rates exhibited higher rates of tooth loss. Furthermore, municipalities with greater coverage by dental specialty centers had lower rates of tooth loss among adolescents. Additionally, female adolescents, older individuals, those whose mothers were less educated, those from low-income households, and those with self-reported dental care needs exhibited a higher frequency of tooth loss. These findings highlight significant and relevant inequities in oral health outcomes.

Adolescence is characterized by physiological, psychological, and social changes.^1^ This developmental stage is critical for health, as adolescents are vulnerable to socioeconomic risk factors and, consequently, more likely to adopt unhealthy behaviors that can persist throughout life, including smoking and poor oral hygiene practices.^1^ Evidence from the literature has shown that tooth loss is strongly associated with a low quality of life,^3^ affecting individuals’ daily activities, as well as their social, emotional, and psychological well-being.^4^

Therefore, understanding tooth loss in adolescence can aid clinical decision-making and the evaluation of interventions, services, and programs, especially in populations that require treatment such as oral and regular health care.^5^

In this study, female adolescents had higher rates of tooth loss compared to male adolescents, which is consistent with the literature.^3,6,7^ One possible explanation is that women report a higher prevalence of oral health problems, exhibit greater self-criticism related to dental appearance, and experience lower self-esteem, in addition to utilizing dental services more frequently.^5,6,8^ Studies conducted in Brazil support this hypothesis, revealing an association between the number of teeth extracted due to dental caries and inadequate access to specialized dental services, such as endodontic treatment, in public health settings. Consequently, tooth extraction becomes the default treatment for advanced caries.^9,10^

Self-perception of dental care need was strongly associated with tooth loss. According to the literature, such perception is shaped by a variety of factors, including individual and personal experiences, vulnerable family environments, adverse social contexts, and poor living conditions that contribute to how individuals perceive their oral health.^14,15^Malocclusion, concerns about appearance, and untreated caries are the most common factors associated with self-reported dental care needs.^15^

There was an association with age, as greater tooth loss was observed among older adolescents, in line with the literature,^6,7^ which demonstrates the cumulative behavior of the outcome, even in adolescents. In addition, the literature has shown that delayed access to oral health services may determine the progression and severity of oral diseases, resulting in the need for mutilating procedures such as tooth loss.^6,7^

Inequities such as low maternal education and low household income were associated with tooth loss. Household income may be an indicator of accumulated knowledge, which can influence the adoption of healthy habits and improvement in social conditions.^6,8,9^ The literature shows a social gradient in tooth loss: the lower the income and education, the higher the tooth loss. Poorer and less educated individuals live in areas with lower supply of fluoridated water, inadequate access to dental services, and unhealthy dietary and hygiene practices that contribute to higher caries prevalence and, consequently, tooth loss.^6^

Additionally, economic constraints are closely associated with the type of dental treatment received. While lower-income individuals are more likely to undergo tooth extractions, those with higher incomes tend to seek routine check-ups and conservative dental treatments, resulting in a higher number of preserved teeth. Our study confirmed that regular check-ups serve as a protective factor against tooth loss, as widely demonstrated in the literature.^14^

Two contextual variables were associated with tooth loss. Municipalities with higher illiteracy rates were associated with a higher prevalence of tooth loss. Evidence from the literature has demonstrated that poorer living conditions are strongly linked to higher rates of untreated caries, tooth loss, and poorer quality of life.^3,5,6,8,9,16^

The National Oral Health Policy (PNSB), published in 2004, expanded and qualified the delivery of specialized dental services through the implementation of dental specialty centers, designed to provide comprehensive oral health care, including specialties such as endodontics and prosthodontics, which are essential for the treatment and rehabilitation of teeth with extensive carious lesions, thus offering viable alternatives and serving as a protective factor against tooth loss, as seen in this study.

Our study also shows that it is essential to improve oral health access and coverage at the local level and to promote more equitable and regular dental services.

This study has some limitations that should be acknowledged. The cross-sectional design does not allow establishing causal relationships between exposure and outcome. To minimize any potential information bias, all contextual variables were collected during the same time period. The study was carried out in 2012 and all the information are related to that year and/or to 2010 (SBBrasil 2010).

The main methodological strengths of this study include the use of a large representative population-based sample from Minas Gerais. Additionally, the high level of reliability of dental examiners reduced the risk of information bias. The considerable sample size allowed for sufficient power to detect associations. Multilevel modeling enabled the assessment of contextual and individual factors, offering a more comprehensive understanding of tooth loss among adolescents. Statistical modeling was based on an adapted theoretical framework on the social determinants of health proposed by the World Health Organization, which was very useful in selecting appropriate variables.^14^

Simultaneously evaluation of contextual and individual indicators of tooth loss among adolescents can help elucidate the extent to which oral health problems are experienced by individuals in different social contexts. Furthermore, an important clinical implication of this study is that oral health promotion strategies aimed at reducing the prevalence of untreated caries have the potential to contribute to reducing tooth loss among adolescents.

From a health policy perspective, actions that promote comprehensive and interdisciplinary care for adolescents and a holistic understanding of the individual are important for reducing tooth loss among adolescents. Finally, intersectoral public policies aimed at reducing social inequalities should be on the agenda of policymakers and stakeholders to improve the quality of life and well-being of adolescents. It is important to mention that, although some progress has been made, intersectoral policies are not effective in practice. Brazil is a country of continental dimensions, and this is associated with significant economic, social, and epidemiological inequalities. Intersectoral policies should be comprehensive and context-specific, aiming to increase families’ access to oral health services and improve their quality of life.

## Conclusion

Among individual variables, demographic characteristics such as female sex and increased age, as well as socioeconomic characteristics such as low household income and low maternal education were associated with tooth loss among adolescents from Minas Gerais, Brazil. In terms of oral health variables, self-perception of oral health was associated with tooth loss, while illiteracy was identified as a significant factor among contextual variables. Protective factors against tooth loss were associated with oral health variables, such as the utilization of dental services, and with contextual variables, such as the presence of secondary dental health care in the municipality. Public policies targeted at the oral health needs of this population are crucial for reducing inequities.

## Data Availability

The datasets generated during and/or analyzed during the current study are available from the corresponding author on reasonable request.

## References

[1] Freire MC, Nery NG, Jordão LM, Abreu MH (2019). Individual and contextual determinants of dental pain in adolescents: evidence from a national survey. Oral Dis.

[2] Davoglio RS, Aerts DR, Abegg C, Freddo SL, Monteiro L (2009). Factors associated with oral health habits and use of dental services by adolescentes. Cad Saude Publica.

[3] Kassebaum NJ, Bernabé E, Dahiya M, Bhandari B, Murray CJ, Marcenes W (2014). Global burden of severe tooth loss: a systematic review and meta-analysis. J Dent Res.

[4] Bernabe E, Marcenes W, Hernandez CR, Bailey J, Abreu LG, Alipour V (2020). Global, regional, and national levels and trends in Burden of Oral Conditions from 1990 to 2017: a systematic analysis for the Global Burden of Disease 2017 Study. J Dent Res.

[5] Peres KG, Cascaes AM, Leão AT, Côrtes MI, Vettore MV (2013). is [Sociodemographic and clinical aspects of quality of life related to oral health in adolescentes. Rev Saude Publica.

[6] Barbato PR, Muller Nagano HC, Zanchet FN, Boing AF, Peres MA (2007). Tooth loss and associated socioeconomic, demographic, and dental-care factors in Brazilian adults: an analysis of the Brazilian Oral Health Survey, 2002-2003. Cad Saude Publica.

[7] Peres MA, Barbato PR, Reis SC, Freitas CH, Antunes JL (2013). Tooth loss in Brazil: analysis of the 2010 Brazilian Oral Health Survey. Rev Saude Publica.

[8] Gerritsen AE, Allen PF, Witter DJ, Bronkhorst EM, Creugers NH (2010). Tooth loss and oral health-related quality of life: a systematic review and meta-analysis. Health Qual Life Outcomes.

[9] Eklund SA, Burt BA (1994). Risk factors for total tooth loss in the United States; longitudinal analysis of national data. J Public Health Dent.

[10] Alves LS, Susin C, Damé-Teixeira N, Maltz M (2014). Tooth loss prevalence and risk indicators among 12-year-old schoolchildren from South Brazil. Caries Res.

[11] World Health Organization (1997). Oral health surveys: basic methods.

[12] Pinto RS, Leal DL, Santos JS, Roncalli AG (2018). Projeto SB Minas Gerais 2012: pesquisa das condições de saúde bucal da população mineira: métodos e resultados. Arq Odontol.

[13] Solar O, Irwin A (2010). A conceptual framework for action on the social determinants of health.

[14] Dhanasekaran R, Nayar S (2015). Self-perceived need for dental care. J Pharm Bioallied Sci.

[15] Lopes RT, Neves ET, Dutra LC, Firmino RT, Lima LC, Paiva SM (2024). Individual and contextual factors associated with adolescents' self-perceived need for treatment. Int J Environ Res Public Health.

[16] Seerig LM, Nascimento GG, Peres MA, Horta BL, Demarco FF (2015). Tooth loss in adults and income: systematic review and meta-analysis. J Dent.

